# Supplementary material for: The Effect of Reduced Fibrinogen on Cerebrovascular Permeability during Traumatic Brain Injury in Fibrinogen Gene Heterozygous Knockout Mice
Source: Biomolecules. 2024 Mar 22;14(4):385. doi: 10.3390/biom14040385 (PMC11048347; doi:10.3390/biom14040385)
Supplement: Supplementary file 1 [file biomolecules-14-00385-s001.zip › biomolecules-2890659-supplementary.pdf]

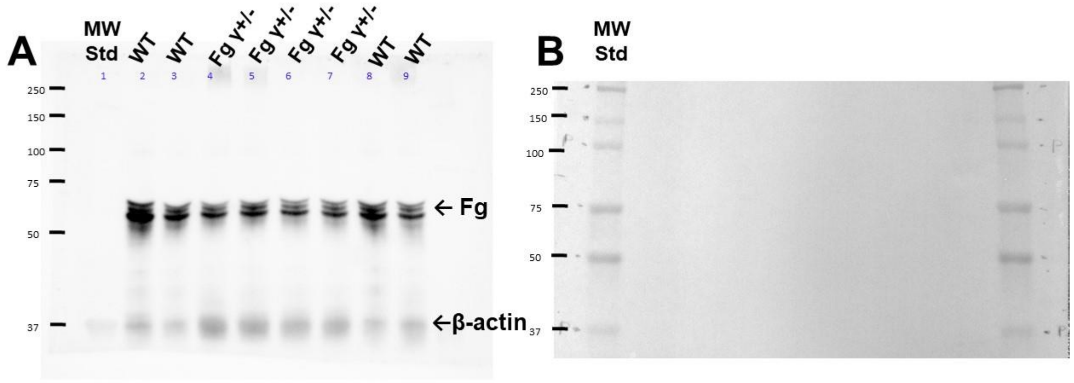

Figure S1. Original Western blot image of Figure 1a with marked (**A**) Fg and (**B**) β-actin molecular weight standard.

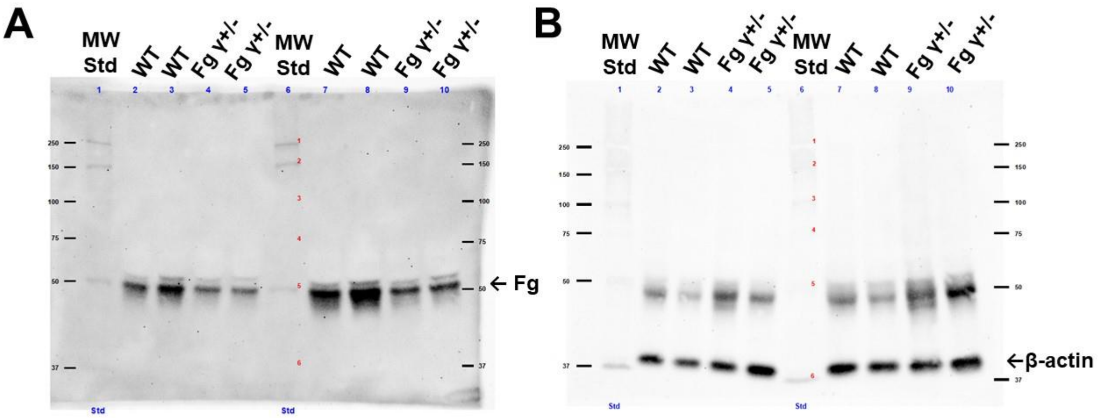

Figure S2. Original Western blot image of Figure 1c with marked (**A**) Fg and (**B**) β-actin.

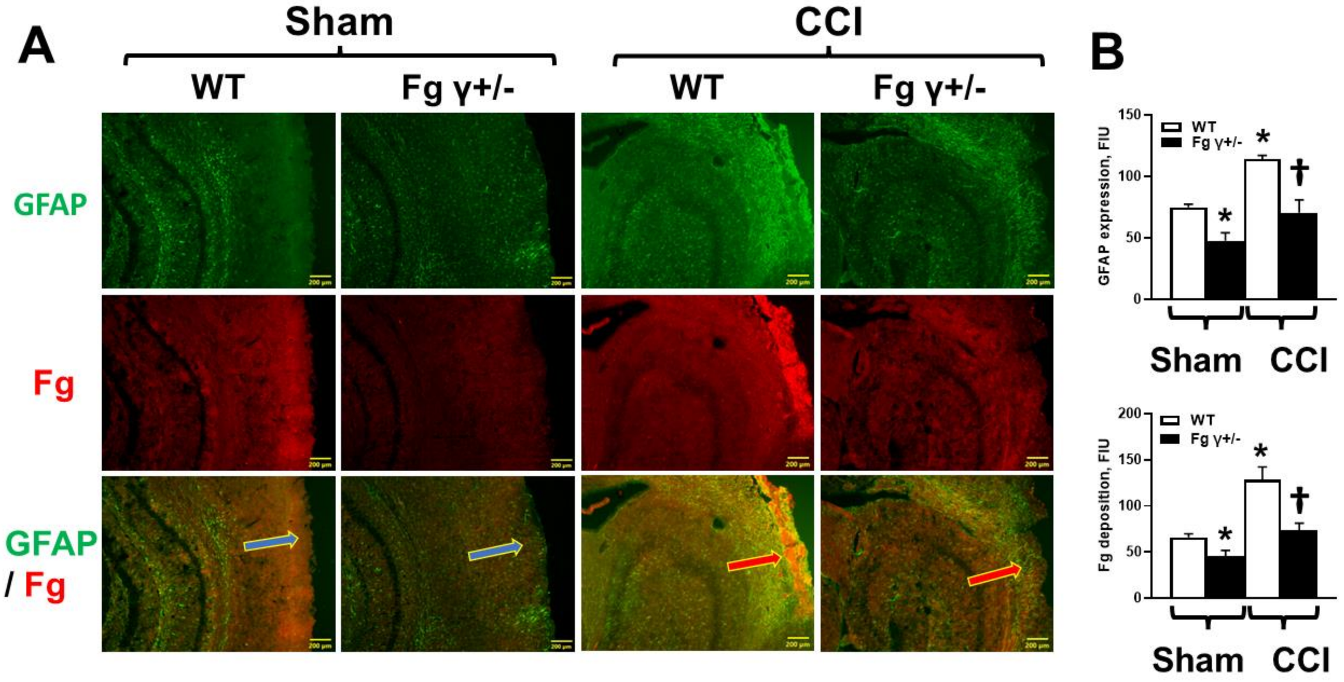

Figure S3. Expression of glial fibrillary acidic protein (GFAP-green) and deposition of fibrinogen (Fg-red) in ipsilateral brain cortical sections 14 days after cortical contusion injury (CCI) or sham-operation (Sham). (**A**) Representative images show marked increases in the expression of GFAP and the deposition of Fg in the cortex of wild-type (WT) mice with CCI in comparison to those in the Fg gamma-chain-knock out (Fg γ+/-) mice after injury. Red arrows show the pericontusional area in injured animals while blue arrows indicate similar location in sham-operated mice. (**B)** Summary of GFAP and Fg fluorescence intensity values in the WT and Fg γ+/- after CCI and sham-operation. p<0.05 in all; ∗ - vs. WT Sham; †- vs. WT CCI; n=4.

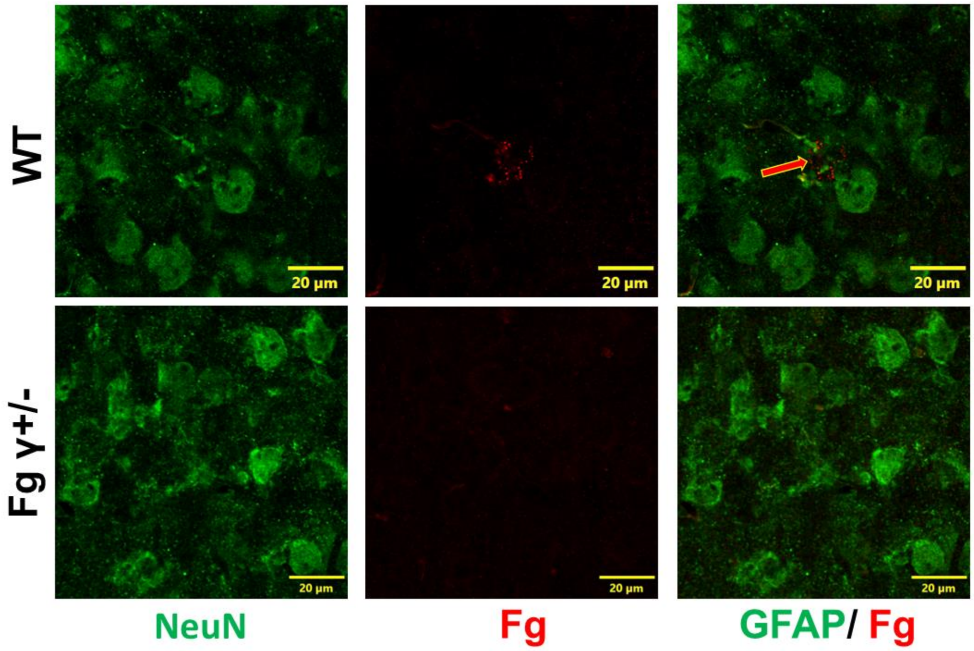

Figure S4. Expression of neuronal nuclei (NeuN-green) and fibrinogen (Fg-red) in ipsilateral brain cortical sections 14 days after cortical contusion injury (CCI). The red arrow indicates deposition of Fg in areas away from the neuron's body.
